# Characterizing dehydration in short-term spaceflight using evidence from Project Mercury

**DOI:** 10.1038/s41526-024-00374-8

**Published:** 2024-06-11

**Authors:** Robert J. Reynolds, Mark Shelhamer, Erik L. Antonsen, William R. Carpentier

**Affiliations:** 1 KBR Services, LLC, Houston, TX USA; 2 The Johns Hopkins University School of Medicine, Baltimore, MD USA; 3 Baylor College of Medicine, Houston, TX USA

**Keywords:** Anatomy, Medical research

## Abstract

Short-term spaceflight is commonly perceived as posing minimal risk to human health and performance. However, despite their duration, short-term flights potentially induce acute physiological changes that create risk to crews. One such change is dehydration (primarily body water loss) due to a heat-stressed environment. Such loss, if severe and prolonged, can lead to decrements in performance as well as increase the risk of more serious medical conditions. Though the general mechanisms of dehydration are broadly understood, the rate and extent of dehydration in short-term spaceflight has not been characterized. Combining data from the six spaceflights of the US Mercury program with a causal diagram illustrating the mechanisms of dehydration, we fit a path model to estimate the causal effects for all pathways in the causal model. Results demonstrate that Mercury astronauts experienced some degree of dehydration across the range of suited time and that the relationship between suited time and dehydration appears to be logarithmic. We discuss causal interpretations of the results and how the results from this and similar analyses can inform countermeasure development for short-term spaceflight.

## Introduction

Short-duration spaceflight was common in the early days of human space exploration but was quickly supplanted by longer-duration spaceflight as space agencies improved technology and increased capabilities. Recently, companies such as SpaceX, Virgin Galactic, and Blue Origin have begun to offer “space tourism” opportunities, making short-duration spaceflight common once again. This offers the opportunity to rekindle the discussion about the acute effects of space travel on human physiology and how much risk those effects may pose to short-duration spaceflight participants. This is of particular concern as space tourists are not professional astronauts and thus lack the benefit of the medical selection and monitoring conducted by governmental space agencies.

One potential set of physiological consequences of short-term spaceflight is fluid shifts and water loss (colloquially known as dehydration). While there is a lack of universal consensus as to the definition of dehydration, for purposes of this study we will use this term interchangeably with body water loss^1^. Water loss is not necessarily a serious medical condition per se, but it can lead to decrements in performance, modulate the effectiveness of pharmaceuticals, as well as increase the risk of more serious medical conditions. While the mechanism for dehydration is generally understood in terrestrial situations, the rate and extent of dehydration in the first 48 h of spaceflight has not been systematically investigated^2,3^. The period immediately after launch is not well characterized by experimental measurements in space but appears to be a time of dynamic fluid shifts both within the intravascular space as well as between the intravascular and extravascular spaces. The sequestration of fluid in extravascular spaces when entering microgravity, would serve to reduce plasma volume but not body weight. According to Norsk, each leg moves approximately 1 L of fluid headward in the body upon entering spaceflight^4–6^. Astronauts typically experience facial fullness that has often been attributed to the development of edema and the characteristic time for this development is not well-known. Norsk does note that on return to Earth visible edema resolves within several hours of landing. During the Space Shuttle era, Leach et al. collected data on body mass and total body water, glomerular filtration rate, and other relevant parameters. However, they did not start taking these measurements until flight day two, which is much longer than all but one of the Mercury astronauts’ flights^5^. Given the significant increase in suborbital and short-duration orbital spaceflight for commercial purposes today, insights gained from the Mercury data are highly relevant to present-day experiences.

The health concerns of short-duration spaceflight are best addressed using data collected in conjunction with actual spaceflight. However, given the difficulty in obtaining high-quality biomedical data during spaceflights of any duration, such data are scarce. There is, therefore, a corresponding need to extricate the most information possible from existing spaceflight data, even those from the early space programs. Among the early missions of the United States space program, the most comparable to modern space tourism—at least in terms of flight duration—are spaceflights from Project Mercury.

Project Mercury was the first human spaceflight program of the National Aeronautics and Space Administration (NASA) in the United States. The program started in October 1958, with its main objective to put an astronaut in orbit and return him safely to Earth. Secondary to this objective was that of evaluating human performance and functional capabilities in space^7,8^. The crewed spaceflights in Project Mercury were of short duration, with the shortest orbital flights lasting just over 4.5 h and the longest lasting just over 34 h^8^. The Mercury spacesuits had poor ventilation and may have created a heat-stress environment for astronauts. They were multilayer suits with neoprene-coated nylon fabric and an outer layer of aluminized nylon^9^.

The biomedical data from Project Mercury are relatively few, as six astronauts completed one flight each during the program, and few medical parameters were measured. Basic physiological measurements were taken on the Mercury astronauts before and after each of the flights, and the total duration of various environmental exposures (suit time, orbital time) was recorded. Despite the small number of observations and variables, when thoughtfully analyzed and carefully interpreted, these data can provide valuable insights for modern spaceflight.

In this study, we analyzed data from Project Mercury to investigate dehydration in short-term spaceflight. To do so we drew a causal diagram of the dehydration process during Mercury flights, then used a path model to estimate the causal effect of time spent in the Mercury pressure suit on four dehydration indicators. As a comparison to the path model, we fit companion linear regression models. We discuss the findings and their external validity, as well as discuss how this and similar studies can be used to aid countermeasure selection. Thus, while this work does not advance our understanding of dehydration generally, it does add to our understanding of the short-term effects of spaceflight.

## Results

### Casual diagram

**Fig. 1:**
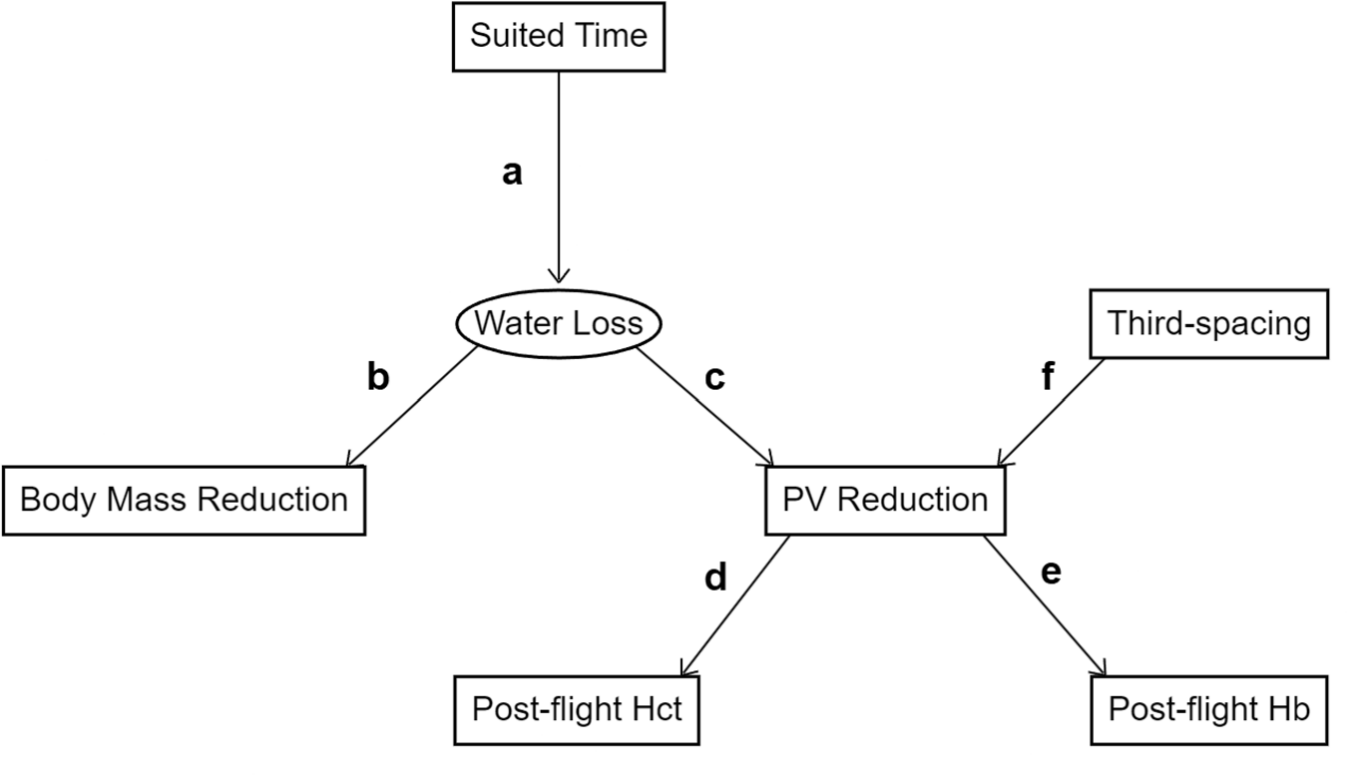
Assumed causal diagram for dehydration during Mercury flights. PV reduction plasma volume reduction, HCT hematocrit, Hb hemoglobin.

Figure 1 presents our causal diagram for dehydration during the Mercury spaceflights, drawn as a Directed Acyclic Graph (DAG). Our diagram contains five observed variables (rectangles) and two latent variables (ovals). The arrows on our DAG indicate that while *Suited time* is the immediate cause of only one variable (*Water loss*), it is the ultimate cause of all other variables except the latent variable *Third-spacing*, since a directed path may be traced from *Suited time* to any of these other variables. *Water loss*, one of the latent variables in this study, represents the amount of water in the body. It is the direct cause of the observed variable *Body mass reduction* and the variable *PV reduction. PV reduction* is plasma volume reduction in the blood, in response to *Water loss* from the body or *Third-spacing* due to posturing and reduced gravity. It is the direct cause of the observed blood composition measures: *Post-flight HCT* and *Post-flight Hb*.

### Correlation matrix

**Fig. 2:**
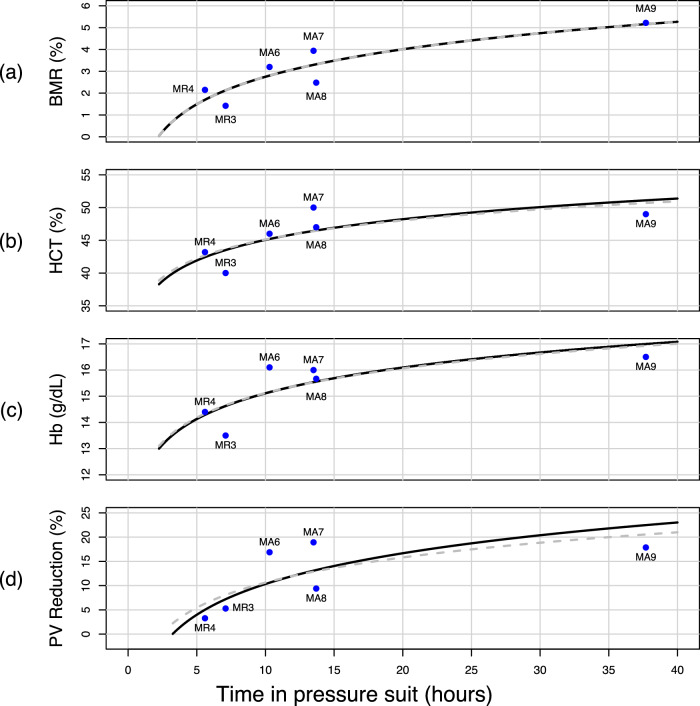
Observed data points and model predictions for the total effect of suited time on dehydration outcomes. **a** Body mass reduction (%); **b** HCT (%); **c** Hb (g/dL); **d** PV reduction (%). In each plot, points represent individual values obtained after the various Mercury flights; solid lines are the estimated values derived from the path models; dashed lines represent estimated values obtained through the linear regression models.

Table 1 presents the correlation matrix between the variables in the Mercury dataset, including the calculated values for *PV reduction*. Here we express suited time using the natural logarithm, as scatterplots suggested that the relationship between suited time and the outcomes was potentially logarithmic (see Fig. 2 below). Since the base of the natural logarithm is Euler’s number (~2.718), log units of suited time are multiples of 2.718 h spent in the pressure suit. One log unit of time would thus be e^1^ = 2.718; 2 units of log time would be e^2^ = 7.389 h, etc.

**Table 1:** Correlations between measured variables

	Body mass reduction	HCT	Hb	PV reduction	Third-spacing
ln(suited time)	0.887	0.748	0.780	0.728	0.390
Body mass reduction		0.856	0.868	0.855	0.495
Post-flight HCT			0.929	0.840	0.606
Post-flight Hb				0.876	0.655
PV reduction					0.874

*ln* natural logarithm, *HCT* hematocrit, *Hb* hemoglobin, *PV* plasma volume.

The values in Table 1 indicate that most variables in the dataset were strongly correlated, with all values between causally related variables being 0.606 or larger. The largest correlations were between the *Post-flight HCT* and *Post-flight Hb* (0.929), followed by *Suited time* with *Body mass reduction* (0.887). Consistent with their assumed non-causal relationship, the lowest correlation was that between *Suited time* and the *Third-spacing*, at 0.390. The correlations between *Suited time* and all blood-related outcomes were also lower than those between *Suited time* and *Body mass reduction* or between the set of blood-related variables with each other.

### Path coefficient estimates

Table 2 displays the estimated path coefficients for the paths in Fig. 1. The estimated path coefficients were all positive and ranged between 0.625 and 1.000. Among the set of simple (single-letter) path coefficients, the largest was estimated for the paths between *Water loss* and both *Body mass reduction* and *PV reduction* (paths b and c in Fig. 1 and Table 2), where the relationship was 1-for-1: for every 1 SD increase above the mean in *Water loss*, there was a corresponding 1 SD increase above the mean in *Body mass reduction* and in *PV reduction*. The smallest estimated simple path coefficient was that connecting *Third-spacing* to *PV reduction* (path f in Fig. 1), with a value of 0.652.

**Table 2:** Path model coefficients corresponding to the causal model of Fig. 1

Path	Cause	Effect	Estimated	Observed
a	ln(suited time)	Water loss	0.887	
b	Water loss	Body mass reduction	1.000	
c	Water loss	PV reduction	1.000	
d	PV reduction	HCT	0.895	0.840
e	PV reduction	Hb	0.909	0.876
f	Third-spacing	PV reduction	0.652	0.874
ab	ln(suited time)	Body mass reduction	0.887	0.887
ac	ln(suited time)	PV reduction	0.891	0.728
acd	ln(suited time)	Post-flight HCT	0.812	0.748
ace	ln(suited time)	Post-flight Hb	0.814	0.780
cd	Water loss	Post-flight HCT	0.916	
ce	Water loss	Post-flight Hb	0.918	
fd	PV reduction	Post-flight HCT	0.625	0.606
fe	PV reduction	Post-flight Hb	0.636	0.655

As we might expect, given their close physiological relationship (and strong correlation), *Post-flight HCT* and *Post-flight Hb* had nearly identical path coefficients linking them to *PV reduction* (paths d and e). They also had essentially identical compound paths leading to them from *Suited time* (acd and ace) at 0.812 and 0.814.

### Linear regression models

The linear regression model coefficients are displayed in Table 3, along with the RMSE for each model. The model of the effect of *Suited time* on *Body mass reduction* estimates that the body mass loss is 1.82% for each multiple of 2.718 h of time spent in the pressure suit. The RMSE of 0.576 means the average prediction error of the model over the original dataset was 0.576% of an astronaut’s preflight body mass.

**Table 3:** Linear regression coefficients for total-effect models of suited time on dehydration outcomes

Model	Intercept	ln(suited time)	RMSE
Body mass reduction (%)	−1.4	1.8	0.576
HCT (%)	35.5	4.2	2.261
Hb (g/100 ml)	12.0	1.4	0.663
PV reduction (%)	−6.6	7.5	4.282

The model for the total effect of *Suited time* on *Post-flight HCT* had an intercept of 35.5%, with an increase of 4.2% for each multiple of 2.718 h in the pressure suit. The model generated a RMSE of 2.261.

The model for *Post-flight Hb* had an intercept of 12.0 g/100 ml, with an estimated increase of 1.36 g/100 ml for each additional log unit of time. For this model, the RMSE indicated an average prediction error of 0.663 g/100 ml. Finally, the regression model for the effect of *Suited time* on *PV reduction* related a decrease of 7.5% of preflight plasma volume for each additional log unit of time in the pressure suit, with a RMSE of 4.3%.

The coefficients for *Suited time* in all models in Table 2 express the change in the effect variables after each logarithmic unit of time. For example, 20 h of suited time is approximately equivalent to 3-log time units. Using this value with the coefficient values in Table 3 for *Body mass reduction*, predicts a value of:

1 $$\left(\frac{1.8 \% }{\log {time}\,{unit}}\right)\times \left(3\log {time}\,{units}\right)-1.4 \% =4.0 \% ,$$

interpreted as a 4% reduction in body mass after 20 h of suited time. It is important to note also that because these models were fit on the logarithmic time scale, the zero-intercept on that scale equates to an intercept at 1 h of suited time on the regular time scale, and there is no intercept at zero hour of suited time. This means the model estimates no change in the dehydration outcomes before 1 h of suited time. However, for the blood outcomes, the threshold for change may be larger than 1 h, as the intercepts of these models are below the ranges of HCT and Hb for healthy males. Heuristically this is a plausible restriction, as it suggests changes in plasma volume will likely not begin to manifest until after a minimum amount of time experiencing postural and gravitational changes and/or a minimum threshold of water loss. As suggested by the curves in Fig. 2, the threshold for change may be at ~2.5 h, as this is the point at which the predicted body mass reduction is 0 and the HCT and Hb begin to rise from the lower end of the typical range.

We call attention to the fact that the well-known approximate 3-to-1 relationship between HCT and Hb is roughly preserved in these models. The ratio of the intercept term of HCT to the intercept term for Hb is (35.5/12.0) = 2.96, suggesting that the group means conform to this ratio, while the ratio of the coefficients for the suited time between models is (4.2/1.4) = 3.0, demonstrating that the rate of change with suited time also conforms to this ideal ratio. The ratio of the predicted values from these models preserves this relationship even better, with a value of (35.5 + 4.2*0.916) / (12.0 + 1.4*0.916) = 2.96 at 2.5 h of suited time.

### Comparison of models

Figure 2 shows four plots, one for each dehydration outcome. Each plot shows the original data points (labeled by mission) and two curves of predicted values: one for the path model estimates (black, solid lines) and one for the linear regression model estimates (gray, dashed lines). The path and regression models correspond closely to one another in each plot and fit the original data points well.

Comparing the RMSEs between the path and linear regression models for each outcome quantifies the similarities in model predictive ability through quantification of the “average error” of its predictions. The RMSE for body mass reduction is 0.576 for both models, while for HCT the RMSEs are 2.352 and 2.261 for the path and linear regression models, respectively. For Hb the values are 0.673 for the path model and 0.663 for the linear regression. The models for *PV reduction* show the most divergence both visually in Fig. 2 and through their RMSEs of 4.405 for the path model vs. 4.282 for the linear regression. Overall, the highly congruent RMSE values and the overlapping tracings in Fig. 2 both point to models that estimate the outcomes equally well.

## Discussion

In this study, we estimated the change in several variables caused by time spent in the Mercury pressure suit. The results show that the relationship between suited time and these measures is well-approximated by logarithmic curves, at least from ~2.5 h of suited time up to ~40 h of suited time, as observed in the Mercury flights. This by itself is a useful finding, as it provides information about the rate and the limits of change in the predicted dehydration indicators. Specifically, the models suggest that values change quickly initially, then at a diminishing rate, until even large increases in exposure time generate little additional effect. For example, the model for *Body mass reduction* predicts a change in body mass of ~2.8% at 10 h, 4.0% at 20 h, and 5.3% at 40 h. This logarithmic relationship gives the models face validity, since water loss from the body cannot continue indefinitely. Instead, the body likely approaches an asymptote where it has rebalanced body water in response to fluid loss as well as fluid shifts occurring in microgravity^10^. These fluid shifts are represented here as an additional cause of *PV reduction*, as they are thought to explain changes in plasma volume (and thus Hb and HCT values) in the first 24 h after entering microgravity, even in the absence of fluid loss^10^. It should be noted that although the rate of loss of water from the body diminishes with time, this does not mean that the effects similarly diminish.

Exploiting the known relationship between sweating and plasma loss, we were able to estimate the volume of fluid that was removed from the blood but which was not lost to sweating. Using this as the measurement of *Third-spacing* allowed us to estimate a value for path **f** on the causal diagram. The larger magnitude of path coefficient c versus path coefficient f supports the notion that *Water loss* translates more directly to *PV reduction* than does *Third-spacing*. This may be especially true as duration in space increases, as the data indicate that those astronauts who were weightless for 4.5 h had the greatest discrepancy in their estimated and actual plasma reduction, suggesting they had the greatest volumes of fluid in extravascular spaces. Research conducted with astronauts on Space Shuttle flights has demonstrated that the mean plasma volume for astronauts was lowest on flight day 2, and was higher on flight days 7–8, and nearly back to preflight levels by flight day 12^10^. This suggests that after an initial, comparatively large fluid shift the body may re-normalize fluid back to the blood. If so, the lower extravascular plasma volumes observed here may indicate that this re-normalization process could begin as soon as 10 h after the start of weightlessness. Again, this is highly speculative, as the data here are few; more targeted and careful research should be conducted to characterize the process and timeline for these shifts in short-term spaceflight and/or in the first few hours of longer spaceflights.

Looking again to the plots in Fig. 2, it appears that the most change occurs in the first 15 h in the pressure suit, after which time the curves become shallow. For the body mass reduction outcome, the models predict that a reduction in body mass can begin in as little as 2.5 h after donning the pressure suit. In contrast, the models for the blood indicators predict change even in the early period, but the change would not result in values outside the normal (non-dehydrated) range until ~10 h of suited time. From a medical management perspective, this suggests that the period of several hours immediately following donning the pressure suit—before spaceflight even begins—may be the most important for preventing dehydration symptoms overall. It is also during this time that astronauts assume a supine position for launch, beginning the process of fluid shifting within the body, which may contribute to changes in blood composition. By intervening in these early hours, the curves for these outcomes may be either shifted to the right or further flattened. However, it also suggests that there may be a window in which to intervene in the dehydration process between the time when enough sweating, urination, and insensible losses have occurred to create a noticeable loss in body mass, but before those losses induce changes in blood composition and subsequent consequences. This less-direct translation between suited time and the blood indicators is expressed in the path coefficients ac, acd, and ace which are generally lower than those on the path linking *Suited time* to *Body mass reduction*.

Though the model here suggests that the rate of body water loss slows over time, it is important to note that the effects of this water loss do not similarly diminish with time. Instead, the effects of water loss are cumulative and thus dependent on the total amount lost at any given moment, with performance changes observable with as little as 2% total body water loss^11^. However, caution is warranted concerning the interpretation of the log modeled data in-hand, as quantitative outcomes appear similar to the sum of insensible and renal body water losses from terrestrial living over the same duration^12^. In addition, it should be noted that there is biological variability in how individuals respond to body water loss, particularly in factors such as triggers for thirst and sweat rate, among others^13^. Thus, there may also be substantial variation in how astronauts adjust to water loss during spaceflight, as some individuals may respond poorly to small amounts of water loss, while others may not experience noticeable effects even after substantial losses. More data are needed to understand the nature of body water losses during short-term spaceflights. Nevertheless, the possibility of dehydration should be one of many considerations in the design of crew health and performance systems for space vehicles as well as in the concept of operations for short-term spaceflight.

In general, causal diagrams and path models that result from them can be useful for informing the best strategies for the prevention of human health and performance outcomes. Prior to any quantitative considerations, careful analysis of the structure and resulting logic of the causal diagram will delineate the possible points of intervention (prevention or mitigation) along the causal pathways that lead to a given outcome. Equally important, if the diagram has sufficient detail, it can eliminate countermeasure candidates by demonstrating that they are not part of any relevant causal pathways; if so, they would not be able to prevent or modify the outcome in question.

Once the possible methods of intervention are identified, path coefficients can help evaluate them by providing a quantitative basis upon which to compare them. Additional considerations—such as resource constraints, technological capabilities, and difficulty of execution—could then be used to further narrow the list of candidate interventions. Reversing this scheme, potential interventions pre-screened to meet resource constraints could be further differentiated by their role in the causal network and/or their magnitude of effect.

In the relatively simple causal diagram of Fig. 1, the structure of the DAG shows us that to prevent water loss we have two choices: either modify one or both components of the exposure (suited time and suit type) or introduce exogenous countermeasures that can act on *Water loss* directly. Since the suited time is a direct result of mission duration, and since the development of new spacesuits is time-consuming and extraordinarily costly, the introduction of exogenous countermeasures seems the more feasible strategy for programs already in operation.

In this analysis, we interpret our path coefficients to be valid causal effect estimates, i.e., unbiased quantitative measures of phenomena we believe are causally linked. We make this claim only after carefully considering the assumptions required for the validity of both our causal explanation of dehydration and the circumstances which gave rise to the Mercury dataset.

First, we are willing to assume that our causal diagram is generally correct. While there are formal methods for DAG validation^14^, they can also be tested for fit to data through fit statistics generated by path models^15^. However, the small dataset used here, and the presence of latent variables, would render these methods uninformative. Instead, we are willing to accept our explanation as correct because water loss and attendant changes to blood composition is a well-known phenomenon with a mechanism that can be represented accurately at a high level^2,16^.

We are also willing to make necessary assumptions about the Mercury data and how they were generated. Modern causal inference theory identifies several assumptions required in order to estimate valid causal effects^17^: positivity, independence of exposure assignment and outcome, exchangeability, and stable unit treatment value. We believe that the great degree of similarity among the Mercury astronauts and the details surrounding Project Mercury allow us to meet these assumptions. Thus, by virtue of what we believe to be an essentially correct causal diagram and a dataset that we believe meets the assumptions for valid causal inference, we treat our estimates as unbiased causal effect estimates (further discussion of the individual causal assumptions and why we believe the Mercury astronauts satisfy them can be found in the Supplementary Methods).

Though our effect estimates may be treated as validly causal in the context of the Mercury flights, this does not guarantee that the estimates would generalize to all short-term spaceflights. The ultimate exposure variable we used here is exposure time, where that time is spent specifically in the Mercury pressure suit. As the need for better cooling in the space suit was recognized after Project Mercury, later suits, such as those used in the Apollo program, had better cooling capabilities. Even with equivalent suited time, the effect on modeling of wearing a suit with better cooling would be a smaller path coefficient. Thus, the estimate for path a, while valid for the Mercury pressure suit, is not valid for all spacesuits generally.

In the context of space tourism, it is unclear the extent to which our model results are generalizable, as each company offering space tourism has designed their own space suit or pressure suit, none of which are as self-contained as modern spacesuits designed by NASA and other government space agencies. It may, therefore, be useful to use the results presented here as a benchmark, with the understanding that, all else being equal, suits which offer greater cooling capability than did the Mercury pressure suits should generate a slower rate of water loss and those with lesser cooling capability will generate an increased rate.

Another potential threat to external validity is whether the estimates obtained here would apply to non-astronaut individuals. To the extent that the physiological mechanisms of dehydration are universal between people, the path coefficients for paths **b** and **c** should generalize since they quantify the causal effect of *Water loss* on physiological consequences. In this way, the structure of the causal diagram recognizes that, regardless of why *Water loss* is at a specific level, the physiological effects should be the same. For similar reasons, paths d and e should be generalized as well. These assumptions, thus the external validity of these findings, could be confirmed either using additional spaceflight data or data from spaceflight analogs.

The Mercury astronauts were all male, so it is also unknown the extent to which these results would generalize to females. Since the mechanism of dehydration is not sex-specific, the logarithmic shape of the response curve should generalize to women, but the rate of water loss (i.e., the magnitude of the path coefficients) may differ. Differences in body composition, the pattern of sweating, and hormones may all lead to different amounts of sweating among women^18^. Nevertheless, evidence from studies of racecar drivers—who routinely spend as much as 3 h in relatively small vehicle cockpits wearing flame-retardant suits and helmets—suggests measurable but clinically and statistically insignificant differences between men and women in dehydration^19^. Given these uncertainties, future research regarding dehydration in short-term spaceflight should include women to provide clarity.

One limitation of the path model stems from the data to which it is fit. The inclusion of a latent variable on the causal diagram limits the model to expressing path coefficients as standardized partial regression coefficients (i.e., in terms of standard deviations above the mean). While this allows us to understand the causal relationship between measured and unmeasured variables, we are unable to provide predicted values for the unmeasured variable, limiting the utility of the model.

Another limitation is the incomplete explanation for dehydration encapsulated in our sub-graph causal diagram (Fig. 1). Additional variables such as water intake, urine and fecal output, and even the potential effect of the Mercury pressure suit on third-spacing would have direct causal relationships with *Water loss*, and it would be valuable to have estimates of direct effect for them. However, without measurements of these variables we cannot obtain such estimates. It is reasonable to assume that beyond 4.5 h astronauts likely urinated and may have defecated in flight. It is physiologically unreasonable to think that an astronaut did not drink water, eat food, and urinate and defecate during a 34-h flight. The effect of the pressure suit would be the same for all astronauts, and might lead to lower third-spacing than if it were not present. That our path model was a good fit to the data without these measures suggests that these factors were of low variability across the Mercury flights. Nevertheless, the lack of measurements for these factors and others is a limitation of the data that creates a limitation in our analysis.

A limitation that has potential impact on the accuracy of the data is that we do not know what the protocol was for the post-flight blood draws. Differences in posture or phlebotomy technique could create variability in the blood results. In *The Medical Support of Manned Spaceflight*, Dr. Charles Berry, NASA Director of Life Sciences during the Mercury era, noted, “Early missions required only simple provisions for the collection of urine and blood samples”^7^. We speculate that this means samples were taken in the sick bay of (vintage 1940s) aircraft carriers in a manner typical of modern phlebotomy: upright, seated in a chair. No matter the procedure, it likely would have been uniform for all crew as it would have been part of the research protocol. As such we can only hope that differences in measured blood values generated by variance in blood draw procedures are minimal.

Finally, small datasets always represent a limitation in empirical research. Of particular concern is the lack of data for suited times between ~14 and 37 h. Data points in this range of exposure time could reveal that the relationship between suited time and dehydration outcomes is something other than logarithmic, though prior research on dehydration has noted a similar response curve for increases in heart rate during dehydration^18^. The small number of observations also precludes the meaningful use of fit statistics in the path model and potentially leads to a lack of precision in coefficient estimates. The small number of variables means we cannot investigate a more thorough causal system including variables such as those mentioned above. An improved study would include spaceflight data from multiple programs, which would provide variability in these factors, consequently enabling their estimation.

Applying a causal diagram and attendant path model was a strength of our analysis. The causal diagram provided additional information through forming variable-relationship constraints reflective of our prior understanding of water loss and related blood composition changes. Fitting the path model according to those constraints allowed the estimation of path coefficients involving the latent variable. Considering whether the study circumstances meet the requirements for the estimation of valid causal effects forced us to carefully consider the interpretation of the path model and to fully understand its uses and limitations. In short, the extra information infused by causal assumptions allowed us to estimate causal effects for the latent variable and understand the limitations to the external validity of our findings, something the use of linear regression models alone would not have easily allowed.

This research provides a salient reminder that dehydration (as reflected by water loss) is a risk in short-duration spaceflight. It also demonstrates that the response curve for dehydration in Mercury spaceflights had a logarithmic shape, with the dehydration process being tied to the time in the pressure suit rather than the length of the spaceflight per se. The implication of this is that dehydration can start relatively early and progress rapidly once an astronaut encounters a heat-stressed environment.

It is our hope that researchers continue to analyze data from early human spaceflight missions to develop a better understanding of the timing and extent of physiological changes upon entering space. Whereas researchers may have previously been deterred by small sample sizes, “noisy” data, or the absence of key variables, analytic methods such as path analysis can overcome some limitations and offer compelling insights useful in situations such as countermeasure development.

## Methods

### Causal diagram

To represent our understanding of how time in the Mercury pressure suit leads to the observed dehydration outcomes, we drew a causal diagram in the form of a *directed acyclic graph* (DAG). These graphs represent causal factors as nodes and the causal influences between them as arrows connecting them. The “directed” property of DAGs refers to the use of (unidirectional) arrows, while the “acyclic” property prohibits feedback loops. These properties enforce the requirement that causes must precede effects, while the graphical nature of DAGs simplifies the formulation and communication of causal explanations—especially in complex causal systems^20^. We employed a DAG here to both aid our understanding and to facilitate the use of a path model, as detailed below.

After assessing that the Mercury dataset contained an appropriate time-based exposure variable and three indicators of dehydration, we drew our DAG to show the process by which the exposure causes the outcomes, based on our general understanding of dehydration from the medical literature. It is important to note that, because our DAG includes only measured variables and some latent variables that are on the causal pathways between them, our DAG represents a limited sub-graph of a larger DAG for the causal mechanisms involved in dehydration. While additional latent variables could be included on the DAG, to the extent that these variables are exogenous to the system or are additional intermediaries on existing paths, the logic of causal diagraming suggests they would not alter the estimation of the total effect of suited time on dehydration outcomes. As such variables are unmeasured here, estimation of direct effects for them would not be possible. Thus, for the sake of presenting a simplified diagram, we have omitted all such additional unmeasured variables from our DAG.

### Path model

After articulating our causal diagram, we used it as the basis for fitting a path model to our data. This allowed us to estimate path coefficients, quantifying the causal effects of variables on each other in accordance with the causal diagram. Path coefficients can be computed using either the correlation matrix or the variance-covariance matrix between the observed variables. When using the correlation matrix, as we did here, the observed correlation between any two variables is taken to be the sum of all paths between them on the causal diagram, when paths are defined according to the rules defined by Wright^21^. We estimated the path coefficients by solving the set of decompositions as a system of simultaneous equations^21^.

Path coefficients derived from a correlation matrix are standardized partial regression coefficients, expressed in units of standard deviation above the mean^15^. For example, a path coefficient of 0.5 would mean that for each increase of 1 SD above the mean in the causal variable, we would expect to see a 0.5 SD increase above the mean in the effect variable.

### Comparison with linear regression

As linear regression is the typical analytic method used to quantify the change in a dependent variable in response to the change in an independent variable, we used it here as a check on some results of our path model. We fit three linear regression models to the data, regressing each dehydration indicator on time in the Mercury pressure suit. If the models offer comparable fit, their predicted values should be close to one another. We determined this graphically, by plotting fitted curves from both the path model and the linear regressions, as well as through use of the root-mean-square error (RMSE). RMSE is in essence the average prediction error, derived from the differences between predicted values from a model and the observed data. A small value for the RMSE is indicative of a model that is a good fit to the observed data, and small differences between the RMSEs generated by the path model and the linear regression models can be interpreted as a comparable fit. All linear regression models were fit using the R statistical computing package^22^.

### Source data and variables

The data used in this study were collected in conjunction with the six crewed Mercury spaceflights^8^. From that dataset, we used the following variables: time in the Mercury pressure suit, measured in hours; post-flight body mass reduction, as a percentage of preflight mass; post-flight hematocrit, expressed as a percentage of total blood volume; and post-flight hemoglobin, measured in units of g/dL. The time in the pressure suit was considered the exposure of interest, and the other three variables were the measured dehydration outcomes. Table 4 shows the Mercury dataset used here, as reported in ref. ^8^. Refs. ^23–28^ provide original sources for the data contained in ref. ^8^ of the article^23–28^.

**Table 4:** Data from the Mercury spaceflights

Flight	Age at launch (yr)	Suited time (hours)	Body mass pre (kg)	Body mass post (kg)	% Change	HCT pre (%)	HCT post (%)	Hb pre (g/100 dL)	Hb post (g/100 dL)	Post-flight PVR (%)^a^	Post-flight extravascular plasma volume (%)^b^
MR-3	37.5	7.1	76.79	75.70	1.42	**39.0**	40.0	13.0	13.5	5.28	−0.05
MR-4	35.3	5.6	68.27	66.80	2.15	42.5	**43.2**	14.1	14.4	3.27	−4.10
MA-6	40.6	10.3	77.79	75.30	3.20	42.3	46.0	14.3	16.1	16.88	6.57
MA-7	37.1	13.5	69.85	67.10	3.94	44.5	50.0	14.4	16.0	18.92	6.54
MA-8	39.6	13.7	80.19	78.20	2.48	44.0	47.0	15.0	**15.7**	9.39	1.10
MA-9	36.2	37.7	66.68	63.20	5.22	44.3	49.0	14.8	16.5	17.86	1.89

*HCT* hematocrit, *Hb* hemoglobin, *PVR* plasma volume reduction.

^a^Values computed from pre- and post-flight blood measures; see the text.

^b^Estimated using the equation in ref. ^3^; see the text.

Bolded entries are imputed values; see Supplementary Material.

We used the percentage change in body mass for analysis because we cannot infer *Water loss* from a single body mass measurement; the *change* in body mass over a short period of time must be used as evidence of fluid loss. We standardized the loss to each astronaut’s preflight body weight to avoid confounding by body size. In contrast, HCT and Hb have normal (non-dehydrated) value ranges, thus single post-flight measurements can be indicative of some degree of *Water loss* and/or third-spacing on their own.

Following the formulas published in ref. ^29^, we were able to calculate the percentage reduction in plasma volume based on preflight Hb, post-flight Hb, and post-flight HCT. These values were then used as the values for the variable *PV reduction* on the DAG.

A prior study of dehydration due to sweating in ref. ^3^ demonstrated that *PV reduction* can be reliably estimated from *Body mass reduction* alone. Using the regression equation for estimating plasma reduction from body mass reduction in sweating, we calculated the expected values of *PV reduction* for each astronaut based on their observed values of *Body mass reduction*. We then subtracted those expected values from the *PV reduction* values calculated based on blood measures. We inferred the difference to be the volume sequestered in extravascular spaces. These data were used as the measurement of *Third-spacing* in the path model.

To establish that any observed post-flight dehydration was not pre-existing, we confirmed that preflight HCT and Hb values were in the normal (non-dehydrated) reference range for healthy males, and that the preflight values were lower than the post-flight values.

A careful examination of the raw data revealed some potential inconsistencies in the data as originally recorded, which led us to replace three values with imputed ones. Details of this exploratory analysis and imputation are presented in the Supplementary Methods.

Since these data were obtained from a peer-reviewed publication freely available on the internet, and because they pertain exclusively to astronauts who were deceased at the time of our study, this research was exempt from institutional human-subjects review.

### Reporting summary

Further information on research design is available in the Nature Research Reporting Summary linked to this article.

### Supplementary information

Supplemental Methods

Reporting Summary

## Data Availability

All data required for this study are presented in Table 4. Our source for these data is from ref. ^8^.
