# Supplementary material for: Characterizing dehydration in short-term spaceflight using evidence from Project Mercury
Source: NPJ Microgravity. 2024 Jun 11;10:64. doi: 10.1038/s41526-024-00374-8 (PMC11166991; doi:10.1038/s41526-024-00374-8)
Supplement: Supplementary file 1 — Supplemental Methods [file 41526_2024_374_MOESM1_ESM.pdf]

Characterizing Dehydration in Short-Term Spaceflight Using Evidence from Project Mercury

Robert J Reynolds, MS MPH PhD[1]

Mark Shelhamer, ScD[2]

Erik L Antonsen, PhD MD[3]

William R Carpentier, MD

[1]KBR Services, LLC, Houston, TX, USA

[2]The Johns Hopkins University School of Medicine, Baltimore, MD, USA

[3]Baylor College of Medicine, Houston, TX, USA

Supplementary Methods

Data Quality Investigation

The Mercury spaceflight dataset as presented in Carpentier et al. [4] contained pre- and postflight measures of HCT and Hb, as well as pre- and post-flight body mass measures. The small number of observations in this dataset makes data accuracy of the utmost importance. However, these measures were made 60 or more years ago, and we have no original data collection forms against which to validate them. Instead, we checked the feasibility and consistency of the data points by examining their internal consistency.

According to the causal diagram in Figure 1 (which is based on our a priori understanding of the physiology of dehydration) the more time astronauts spent in the Mercury pressure suit the greater the evidence we should see of dehydration. Thus, we would expect, all else being equal, that for all observations in the dataset we should see a reduction in body mass (i.e., that astronauts would lose "water weight"), that post-flight values of HCT and Hb would be greater than pre-flight values, and that all dehydration outcomes would directionally agree. For example, if an astronaut has experienced post-flight body mass reduction, then we would also expect to see some indication of dehydration in his blood composition measures, and vice versa. In addition to this, we employed a quality check on the blood composition measures using a well-known relationship between HCT and Hb: the value of HCT should be approximately 3 times the value of Hb. We thus checked that (1) body mass reduction and blood composition variables moved together; (2) post-flight HCT and Hb values were larger than pre-flight values; and (3) the HCT/Hb ratio was approximately 3.0. (Operationally we considered any ratio to be valid if it was between 2.85 and 3.15, i.e., within 5% of the theoretical value of 3.0.)

Examination of the data demonstrated a positive post-flight weight loss for all astronauts. Thus, we expected to see increases in HCT and Hb in all observations, and this was indeed the case for 3 of the 6 observations. For the MR-3 flight the pre-flight HCT value of 45 was larger than the post-flight value of

40. However, that astronaut experienced weight loss and his post-flight Hb was elevated in comparison to his pre-flight value. A possible explanation of this decrease in HCT is that either the preflight and post-flight values were swapped in the dataset, or the pre-flight datapoint was erroneously recorded. Examination of the HCT/Hb ratios demonstrated that the pre-flight ratio was excessive at 3.46, but the post-flight ratio was within acceptable bounds at 2.96. Taken together, this is suggestive of an error in the pre-flight HCT value and an accurate post-flight HCT value. Ultimately, we deemed the postflight HCT value correct, and thus adjusted the pre-flight HCT to triple the value of the pre-flight Hb, a value of 39, replacing the originally recorded value of 45.0.

The observation for MR-4 also contained a pre-flight HCT value larger than its post-flight value. However, this difference was small (42.5 pre-flight vs. 42.2 post-flight), echoed by a similarly small change in the Hb~~,~~ (14.1 to 14.4), and a small amount of body mass reduction in comparison to the other astronauts~~.~~ (2.1% vs. mean 3.3% for all others). Additionally, his pre- and post-flight HCT/Hb ratios were within acceptable limits~~.~~ at 3.01 and 2.93. Given this information, it seemed most likely to us that the data reflect a minimally changed blood composition for this astronaut after his 5.6 hours of suit time (the least amount of suited time of any of the 6 flights). Nevertheless, for the sake of consistency we adjusted the post-flight HCT observation for MR-4 upward to be three times his post-flight Hb, for a new value of 43.2.

In the observation for MA-8, the Hb appears to have declined over the course of the spaceflight (14.5 post-flight vs. 15.0 pre-flight). This observation led to an out-of-range post-flight HCT/Hb ratio of 3.24. As the changes in HCT were in the expected direction and of reasonable magnitude, and the preflight HCT/Hb ratio was within acceptable bounds (2.93), we judged the post-flight Hb to be potentially erroneous. To impute a new value for post-flight Hb we divided the post-flight HCT value by 3, to satisfy the 3-to-1 ratio. This gave an imputed post-flight Hb value of (47/3) = 15.7.

To gauge the impact of these changes in the dataset, we examined the correlations involving post-flight Hb or post-flight HCT both before and after imputation of the three values. Overall, the shifts in the correlations were minimal as all correlations after imputation were within 10% of their original values. As expected, the correlation between Hb and HCT was strengthened, as we adjusted Hb and HCT values to respect the 3-to-1 relationship between HCT and Hb.

Consideration of Causal Assumptions

In the main text we noted that we believe the linear regression estimates provided in the article may be considered valid causal estimates of the total effect of suited time on the dehydration outcomes, and that the path coefficients may be considered valid causal effect estimates for the direct effects of various variables on each other. We are willing to make this assertion in part because we have fit the statistical models in accordance with what we believe to be an accurate causal model for dehydration. However, just because the causal model is accurate does not mean that the data are able to estimate valid causal effects; biases may still be present in the data themselves.

To assume valid causal estimates, we needed to consider whether we thought the Mercury data met several additional assumptions:

a. **Positivity** is the assumption that all study subjects had a non-zero chance of receiving all exposures. In this context, this means that all six Mercury astronauts under study would have had a non-zero chance of spending from 0 to 38 hours in the Mercury pressure suit during their flights. This is essentially equivalent to saying that each of the astronauts could have been received any flight assignment. Lacking evidence to suggest otherwise, we are willing to assume positivity.

b. **Independence** states that exposure is independent of potential outcomes. Here this means that flight assignments were not determined by anticipated physiological

reactions to time spent in the pressure suit. As we are aware of no historical evidence to suggest that individual potential medical outcomes were ever a consideration in mission assignment, we are willing to assume independence in the Mercury data.

c. **Exchangeability** is the assumption that there are no systematic differences between groups receiving different levels of an exposure, thus exposed groups (astronauts) could be *exchanged* between exposures without changing the study results. While exchangeability cannot be assessed in empirical datasets (13), we nevertheless believe there is a strong argument for exchangeability among Mercury astronauts. Mercury astronauts were selected according to strict medical and anthropometric requirements from a pool of candidates restricted to military test pilots. As a result, the astronauts were all male, all white, between 35 and 40 years of age at time of flight, all college-educated, all military test pilots, had a maximum permissible height of 180 cm (actual mean 177 cm, actual range 170 cm to 180 cm), and conformed to all other stringent medical requirements. In our view this makes all six Mercury flight astronauts analyzed here exchangeable, even down to an individual basis.

d. **Stable Unit Treatment Value (SUTVA)** truly describes two assumptions: that the potential outcome for one study subject does not change depending on the exposure received by other subjects (no interference) and that there are not multiple versions of a given exposure level such that they lead to different potential outcomes (uniformity of exposure). Here too we believe this assumption to be valid, as the astronauts all completed their flights wearing the same pressure suit, in essentially the same spacecraft environments.
